# The Potential Benefits of Dietary Polyphenols for Peripheral Nerve Regeneration

**DOI:** 10.3390/ijms23095177

**Published:** 2022-05-05

**Authors:** Luisa Muratori, Federica Fregnan, Monica Maurina, Kirsten Haastert-Talini, Giulia Ronchi

**Affiliations:** 1 Department of Clinical and Biological Sciences, University of Torino, 10043 Orbassano, (Torino), Italy; luisa.muratori@unito.it (L.M.); federica.fregnan@unito.it (F.F.); monica.maurina@edu.unito.it (M.M.); 2 Neuroscience Institute Cavalieri Ottolenghi (NICO), 10043 Orbassano, (Torino), Italy; 3 Institute of Neuroanatomy and Cell Biology, Hannover Medical School, 30625 Hannover, Germany; haastert-talini.kirsten@mh-hannover.de; 4 Center for Systems Neuroscience (ZSN), 30559 Hannover, Germany

**Keywords:** nerve injuries, diet, food, flavonoid, non-flavonoid

## Abstract

Peripheral nerves are frequently affected by lesions caused by trauma (work accidents, car incidents, combat injuries) and following surgical procedures (for instance cancer resection), resulting in loss of motor and sensory function with lifelong impairments. Irrespective of the intrinsic capability of the peripheral nervous system for regeneration, spontaneous or surgically supported regeneration is often unsatisfactory with the limited functional success of nerve repair. For this reason, many efforts have been made to improve the regeneration process. Beyond innovative microsurgical methods that, in certain cases, are necessary to repair nerve injuries, different nonsurgical treatment approaches and adjunctive therapies have been investigated to enhance nerve regeneration. One possibility could be taking advantage of a healthy diet or lifestyle and their relation with proper body functions. Over the years, scientific evidence has been obtained on the benefits of the intake of polyphenols or polyphenol-rich foods in humans, highlighting the neuroprotective effects of these compounds in many neurodegenerative diseases. In order to improve the available knowledge about the potential beneficial role of polyphenols in the process of peripheral nerve regeneration, this review assessed the biological effects of polyphenol administration in supporting and promoting the regenerative process after peripheral nerve injury.

## 1. Introduction

Peripheral nerve injuries (PNIs), resulting from trauma or iatrogenic damage [1,2], can be severe and permanently debilitating, affecting over one million people every year worldwide [3]. Despite the meticulous microsurgical repair techniques, including direct repair and auto- or allografting, approximately one-third of all PNIs remain, with incomplete functional recovery that may include total or incomplete recovery of motor and/or sensory function. These conditions can also induce chronic pain, muscle atrophy, and profound weakness, resulting in lifelong disabilities [4] and follow-up costs [5].

Approaches complementary to surgery have been investigated and are still under investigation in order to increase our understanding of their potential role in enhancing nerve regeneration. Nonsurgical treatments include physical exercise, electrical stimulation, phototherapy, laser therapy, delivery of growth factors, cell-based therapies, secretome release, gene therapy, and administration of other pharmacological agents. For many of these approaches, clinical implementation is still far away because of different limitations, as recently reviewed by [6,7]. A complementary and safe approach for supporting peripheral nerve regeneration can derive from dietary adaptations: comprehensive review articles have recently focused on the respective supplementation of macro- and micronutrients [8], or many different plants and herbs [9].

Recently, the role of polyphenols in supporting and modulating the regenerative process after injury of different tissue has been intensively investigated considering their beneficial effects as antioxidant and anti-inflammatory molecules. Polyphenols are a class of bioactive compounds widely distributed in the plant kingdom, which are a part of the food chain, with the ability to interact with living tissues [10]. Therefore, polyphenols could be good candidates as complementary diet substitutes for supporting peripheral nerve regeneration [10,11,12,13,14].

Since the development of neuropathic pain can be related to a previous peripheral nerve injury [15], the aim of this narrative review is to provide a comprehensive update of the literature in order to improve the knowledge about the potential beneficial effects of polyphenols on peripheral nerve regeneration.

## 2. Polyphenols Classification

**Figure 1:**
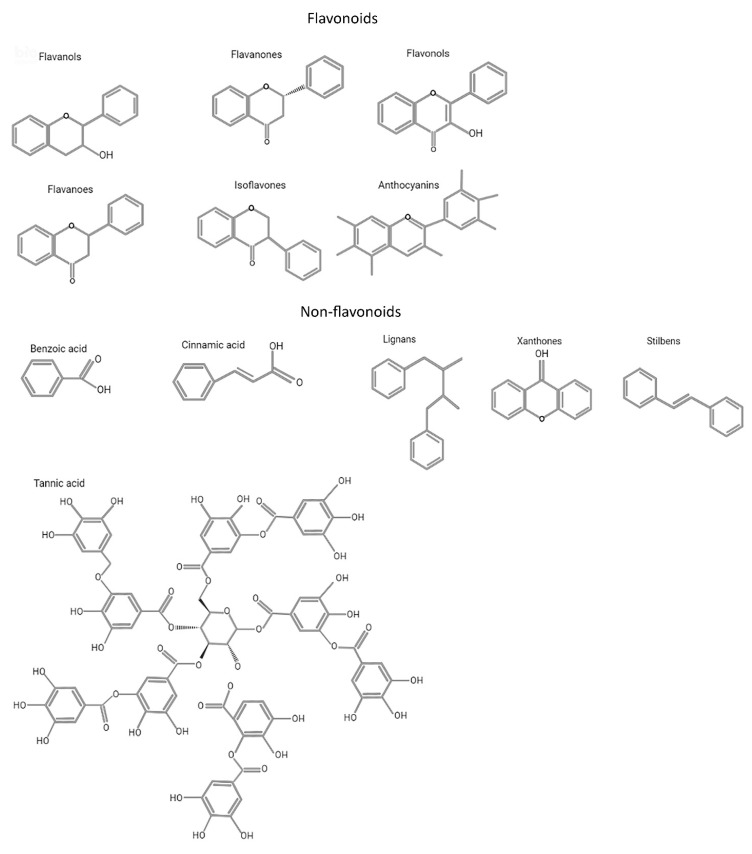
Chemical structures of major groups of polyphenols.

Polyphenols exist in a multitude of variable structures resulting from the various possibilities of adding and combining aromatic rings and hydroxyl groups. Figure 1 shows the most relevant chemical structures of major groups of polyphenols. Polyphenols are found in hundreds of edible plants, fruits, and beverages, such as tea, coffee, and red wine, but even vegetables, leguminous plants, and cereals represent sources [16]. The presence of polyphenols in food not only influences its taste and color but also its nutritional properties and value [17].

The variety of combinations of functional groups (hydroxyls, alcohols, aldehydes, alkyls, benzyl rings, steroids) within polyphenols leads to a great diversity among their chemical structures and specific characteristics (Figure 1). Indeed, within nutraceutical compounds, more than 8000 different phenolic compounds have been identified in the plant kingdom, representing one of the widest classes of plant secondary metabolites [18].

**Figure 2:**
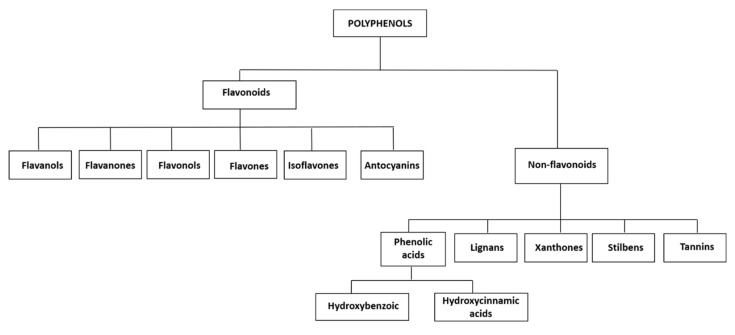
Polyphenols classification.

Figure 2 shows a pedigree chart of the classification of polyphenols into their main groups and subgroups. There is no accordance in the literature about a common classification of polyphenols, the most adopted classification, however, uses a main subdivision into two subgroups: flavonoid and non-flavonoid polyphenols [17] (Figure 2). Flavonoids can be classified into several subclasses: flavanols, flavanones, flavonols, flavones, isoflavones, and anthocyanins (Figure 2). Non-flavonoid polyphenols in foodstuffs include phenolic acids (hydroxybenzoic and hydroxycinnamic acids), lignans, xanthones, stilbenes, and tannins (Figure 2) [19,20]. Free phenolic acids can be found in many fruits and vegetables, whereas bound phenolic acids are most common in grains and grain derivatives [16].

In the following, we review the information available for the different main groups and subgroups of polyphenols, as classified in Figure 2. For each in vivo study available in the literature, we present details on the peripheral nerve lesion model used, the administration roots of the evaluated polyphenolic compound, and the experimental groups in a table at the end of the respective section. We took advantage of the possibility to describe the evaluated parameters and the specific study outcomes in more detail in the text.

## 3. Flavonoids

### 3.1. Flavanols

Flavanols are mainly found in tea, apples, beans, almonds, pistachios, and red wine. They are primarily distributed into four levorotary enantiomers (-): (−)-epicatechin (EC), (−)-epicatechin-3-gallate (ECG), (−)-epigallocatechin (EGC), and (−)-epigallocatechin-3-gallate (EGCG). EGCG is the most abundant and major polyphenolic compound found in green tea, that, with a total average of 65% catechin content, is the main contributor to most of the therapeutic phenomena exerted by green tea [21,22]. EGCG is known to display multiple actions, such as free radical scavenging, inhibition of oxidative stress, and modulating apoptosis events, and is known to evolve pro-oxidative, anti-inflammatory, anticancer, antimutagenic, chemopreventive, angiogenetic, anticholesterolemic, and antiobesity properties. The consumption of EGCG has been also shown to exhibit many neuroprotective effects and to improve cognitive function and learning ability [22,23].

Table 1 summarizes methodological details of studies that evaluated the effects of flavanols (specifically EGCG and green tea extracts) on peripheral nerve regeneration after different types of injuries. The main findings of the respective studies are presented in the following text.

Rats systemically treated with EGCG after sciatic nerve crush injury showed faster motor (foot position, toe spread, extensor postural thrust, and hopping tests) and sensory (reduction in mechanical allodynia and hyperalgesia latencies and improvement in recovery from nociception deficits in heat withdrawal and tail flick withdrawal latencies) recovery, as revealed by [24,25,26]. (See Table 1 for details on model, administration, and experimental groups.) This was related to a protective effect on skeletal muscle fibers, which were prevented from cellular death by activation of antiapoptotic signaling pathways (normalization of the Bax/Bcl-2 ratio and inhibition of the overexpression of the p53 apoptotic gene) and demonstrated an improved recovery of skeletal muscle morphological parameters [24]. Furthermore, a morphological reduction in nerve degeneration [27] and an improved axonal and myelin regeneration, as well as improved ultrastructural parameters, were detected in EGCG-treated animals compared with vehicle-treated animals. The latter was demonstrated by normal and healthy unmyelinated and myelinated axons with normal and regular myelin sheath thickness, normal appearance of collagen fibers, absence of disintegrated myelin figures, and the regenerated and more normalized appearance of Schmidt–Lantermann clefts, which were all features detected to be similar to sham group samples [22]. Moreover, regenerated nerves of EGCG-treated animals showed increased myelinated fiber diameter and myelin thickness, with decreased fiber density compared with untreated animals [28].

A neuroprotective effect of EGCG on spinal cord neurons after sciatic nerve crush injury was detected together with its pro-regenerative effect. Both have been proposed to be mediated through the modulation of neurotrophic factors and their receptors, because EGCG treatment significantly increased expression levels of brain-derived neurotrophic factor (BDNF), Glial-cell-derived neurotrophic factor (GDNF), Neurotrophin-3 (NT-3), Tropomyosin receptor kinase B (TrkB), Trk-C, and p75 neurotrophin receptor in the L3-L6 spinal cord, compared with untreated animals [25] (Table 1). The systemic administration of EGCG after nerve crush injury showed restoration of mRNA expression of Bax, Bcl-2, and survivin, but not that of p53 to sham levels [25]. EGCG has also been demonstrated to protect neuronal cells against retrograde apoptosis following nerve transection. Indeed, rats systemically treated with EGCG and subjected to sciatic nerve transection without repair displayed attenuated caspase-3, COX-2 expression, and TUNEL reaction with an increase in S100B expression [29] (Table 1).

Investigating motor neurons projecting into the vagal and the hypoglossal nerve of rats after nerve crush lesion revealed potent antioxidative effects of a systemic pretreatment with EGCG. The treatment reduced neuronal expression of nicotinamide adenine dinucleotide phosphate-diaphorase (NADPH-d)/neuronal nitric oxide (nNOS) and consequently improved survival of the motor neurons in the hypoglossal nucleus and dorsal motor nucleus of the vagal nerve in the brainstem [30] (Table 1). Moreover, EGCG-treated rats demonstrated to experience significantly less generalized oxidative stress (decrease in isoprostanes in the urine and increase in the total antioxidant capacity of the blood). Furthermore, induced expression of glutathione reductase and suppressed induction of heme oxygenase 1 gene expression in the spinal cord, compared with untreated animals after sciatic nerve crush [26], as well as decreased levels of lipid peroxidation [27], have been described (Table 1). After nerve transection without repair, decreased levels of malondialdehyde (MDA) and increased superoxide dismutase (SOD) and catalase (CAT) activities have also been shown [29].

Chu and colleagues [31] developed a collagen membrane cross-linked with different concentrations of EGCG (0.0064%, 0.064%, and 0.64%) and evaluated its effect on a Schwann cell line (RSC96) in vitro. They report a dose-dependent, EGCG-induced Schwann cell proliferation and differentiation. This was indicated by an increase in the expression of Krox-20, nerve growth factor (NGF), and BDNF after EGCG treatment, and a downregulation of reactive oxygen species (ROS) levels and the MAPK P38 signaling pathway [31].

Taken together, the results of these studies support the hypothesis that EGCG treatment is a potent stimulator of regeneration after nerve injury, and this effect seems to be mostly due to its antioxidant activities resulting from reactive oxygen species scavenging, and inhibition of free radical formation and lipid peroxidation.

As written above, EGCG is the most abundant polyphenolic compound found in green tea. However, the polyphenol mix extracted from green tea is not only composed of EGCG, but also of other catechins, in different proportions. Many different studies have therefore investigated the effect of green tea polyphenols (GTP) on peripheral nerves (Table 1).

Peripheral nerve segments kept in a solution with green tea for 7 days and further in DMEM for 21 days were reported to show less degenerative changes and intact basal lamina and cytoplasmic membrane structures together with vital Schwann cells. In contrast, storage in DMEM alone resulted in more evident death of Schwann cells [32]. However, an electron microscopy analysis of a canine sciatic nerve preserved in green tea revealed that Schwann cell structure was preserved only within 500–700 µm depth from the surface. Cells in deeper layers of the nerve were damaged or had disappeared, thus demonstrating an infiltration limit of polyphenol solution into nerval tissue, from which the authors concluded that only nerve segments with a diameter up to 1.0–1.4 mm can be effectively preserved in green tea solution [33]. After implantation of nerve segments stored in GTP to recipient rats for nerve gap repair, regeneration was reported to be improved over repair with DMEM-stored nerve grafts (increased number and size of myelinated fibers); the authors concluded that this kind of storage protects tissue from ischemic damage [32,33,34,35,36]. Additionally, preservation in GTP was described to be more beneficial than pretreatment with irradiation—a physical method proposed to decellularize nerve allografts and minimize the host immune rejection against them—in terms of accelerated motor nerve conduction velocity and an increase in myelinated fibers, Schwann cell proliferation, and newly formed blood vessels [36].

The preservation of nerve allograft in GTP was also reported to reduce the host-versus-graft immune reaction associated with peripheral nerve allotransplantation in rats [34]. However, in a more challenging model (30 mm nerve defect in a canine model), the storage of nerve segments in the polyphenol solution alone did not completely suppress the immune rejection induced by the allotransplantation, and a therapeutic dose of the immunosuppressant tacrolimus was needed for increasing the outcome of nerve regeneration [35].

Intraperitoneal injection of GTP has also been shown to improve nerve regeneration after end-to-end repair of rat sciatic nerve. Rats who received GTP demonstrated less fibrosis, increased nerve conduction velocity and skeletal muscle wet weight, and increased number and size of myelinated fibers associated with higher expression of NGF, GAP-43 (axonal regeneration marker), Neurofilament (NF)200, and myelin-associated glycoprotein (MAG) in the regenerated nerve compared with vehicle-treated rats [37].

### 3.2. Flavanones

Flavanones exist in tomato, potato, peppermint, rosemary, lemon, orange, strawberry, and plum. Flavanones are a class of flavonoids which includes hesperidin and naringenin. Hesperidin exists abundantly in the pericarp of several citrus fruits (oranges, grapefruit, lemon, and tangerines) and has been demonstrated to have antioxidative, anti-inflammatory, analgesic, and antidiabetic effects.

In a very recent study, the effect of Hesperidin on nerve regeneration was evaluated at different concentrations (0.1%, 1%, and 10% (*w*/*v*) loaded into a cross-linked alginate/chitosan hydrogel. The authors first assessed the hydrogels in vitro in terms of porosity, swelling behavior, degradation rate, release profile of hesperidin, hemocompatibility, antibacterial properties, and absence of cytotoxic effects and showed the general suitability of the hydrogel [38]. In vivo, the hydrogel was injected to the site of a sciatic crush injury, and the results showed an increased functional recovery rate (better sciatic functional index, better results in the sensory recovery test, hotplate test, and less muscle weight loss) in the group treated with 1% hesperidin compared with the other experimental groups. Hematoxylin and eosin staining of both regenerated sciatic nerves and reinnervated gastrocnemius muscles showed good morphology of the tissues too. The authors therefore concluded that hesperidin within an alginate/chitosan hydrogel has a potential application in the treatment of peripheral nerve injuries [38]. Although the aforementioned study is the only study in which the effect of hesperidin was investigated on nerve regeneration following trauma (see Table 2 for details on model, administration, and experimental groups), multiple studies have been conducted on its effect in neuropathic pain models, revealing benefits of hesperidin in reducing neuropathic pain (see the recent reviews [39,40]). The exact molecular mechanism of action is yet to be elucidated, but hesperidin is overall thought to mediate its anti-inflammatory properties via downregulation of multiple proinflammatory cytokines. We can hypothesize that this mechanism at least partially also underlies its positive effects on nerve regeneration following trauma.

Naringenin is a flavanone typically found in citrus fruits, herbal teas, potatoes, bergamot, and tomatoes, and is derived from its precursor naringin via hydrolysis; it offers a wide variety of promising features, such as anti-inflammatory, antiapoptotic, antiosteoporotic, anticarcinogenic, antiulcer, and neuroprotective properties. The beneficial effects of naringenin on neuropathic pain have already been demonstrated and recently summarized [39,40]. However, until now, only a few studies have investigated the potential role of this compound on peripheral nerve regeneration (Table 2).

The effect of naringenin was tested by local administration into a sciatic nerve crush injury site after loading the substance into a collagen type I hydrogel [41] or into a cross-linked alginate/chitosan hydrogel [42], respectively. Naringenin treatment resulted in improved functional recovery, demonstrated by better sciatic functional index, improved thermal pain sensitivity, enhanced electrophysiological activity, and less muscle weight loss compared with the group treated with naringenin-free collagen or alginate/chitosan hydrogels [41,42]. Finally, the authors state that hematoxylin and eosin staining of the regenerated sciatic nerve revealed improvements in myelin sheath and fibers condition when naringenin was administrated and the reinnervated gastrocnemius muscles showed lower fibrosis and muscular shrinkage [41,42]. For combined administration of naringenin with berberine, an improved regeneration outcome was reported [42]. Berberine is an alkaloid with potent anti-inflammatory and neuroprotective effects extracted from the rhizome, roots, and stems of different plants [42].

The effect of naringenin on nerve regeneration after crush injury was also tested by systemic administration, alone or complexed with hydroxypropyl-β-cyclodextrin, to increase its solubility and stability. Sensory and motor assessments showed reduced mechanical hyperalgesia and improved sciatic functional index with systemic naringenin treatment. The treatment also showed an anti-inflammatory effect, demonstrated by a decrease in tumor necrosis factor TNF-α and interleukin (IL)-1β and an increase in IL-10 in the lumbar spine. Naringenin treatment was able to inhibit p75 neurotrophin receptor and c-jun N-terminal kinase pathways, leading to a decrease in caspase 3 levels, suggesting the presence of fewer apoptotic cells in the sciatic nerves at 14 days. Finally, morphological evaluation revealed good regeneration that was further improved by the presence of hydroxypropyl-β-cyclodextrin [43].

The well-known antioxidant potential of naringenin could also explain its beneficial effect on nerve regeneration, together with its anti-inflammatory effect. By acting as a non-enzymatic antioxidant defense, naringenin maintains cellular redox homeostasis, and may consequently decrease the damage caused by nerve injury [39,40].

Despite these promising properties, clinical application of both hesperidin and naringenin is limited because they have low solubility and minimal bioavailability due to their hydrophobic nature. Encapsulation of these natural substances into nanocarriers [42] is therefore a good alternative to improve their solubility and bioavailability.

### 3.3. Flavonols

Flavonols are present in apples, onions, broccoli, lettuce, tea, and red wine. Many studies have reported the beneficial effect of quercetin and myricetin in the context of regeneration of the peripheral nervous system.

Quercetin is a very abundant flavonol naturally administered to the body by dietary intake. Its presence as a secondary metabolite in plants in the form of quercetin glycosides [44] results in its wide availability in the human diet through the consumption of vegetables and fruits (e.g., onions and apples). Differently, as a dietary supplement, it contains mostly the free form of quercetin, the aglycone.

Many in vitro and in vivo experiments and clinical studies describe the beneficial biological effects of quercetin. The antioxidative, anti-inflammatory, immunoprotective, and even anticarcinogenic effects of quercetin have been demonstrated, with resulting benefits in cardiovascular diseases, diabetes, inflammation, viral infections, and cancer prevention [45]. In vitro studies attributed to quercetin the role of increasing neuronal survival [46,47] and reducing neurotoxicity and neuroinflammation.

Table 3 summarizes details on peripheral nerve lesion model, administration roots, and experimental groups for in vivo studies on flavonols. The respective study results are described in the following paragraphs.

Wang and colleagues were the first to report about quercetin effects after repair of a 15 mm gap rat sciatic nerve lesion with a rubber chamber filled with increasing concentrations of quercetin dissolved in saline solution [48]. A duration of 8 weeks after surgery, nerve morphometric analysis revealed that the number and density of myelinated fibers were significantly increased in the regenerated nerves of quercetin-treated animals. Moreover, electrophysiological tests revealed a larger amplitude and area of evoked muscle action potential (MAP) in the two groups that received 0.1 and 1 µg/mL quercetin, respectively.

Chen and colleagues provided evidences on improved sensorimotor recovery in mice after sciatic nerve crush after quercetin injection into the plantar muscle once daily. In particular, the high dose of quercetin (20 mg/kg/day) sucessfully promoted the initial and full recovery of motor function in the toes. Another important outcome was represented by a significant upregulation of genes linked to axonal growth, to the cAMP signaling pathway, and to myelin function. Importantly, while plantar muscle atrophy was observed in mice with lesions of the sciatic nerve on the collateral, control, and injured ipsilateral side, quercetin administered at the maximum dose (20 mg/kg/day), as well as applied mouse NGF—used here as a control pro-regenerative factor—significantly reduced muscle atrophy [49].

Qiu and colleagues, who administered isoquercetrin (quercetin-3-glucoside) intraperitoneally, later used the same experimental model. The authors also describe an improved functional motor recovery as revealed by better SFI values and increased CMAP amplitudes in the isoquercetin versus the saline-treated group. Treatment with isoquercetrin further resulted in significantly increased mean axonal diameters and myelin thickness in the regenerated nerves [50].

Another study likewise reported that rats subjected to sciatic nerve crush injury and intragastrically administered with quercetin showed improved nerve regeneration, with axonal structure and myelin sheath morphology closer to normal nerves. The treatment further showed an antiapoptotic effect and a positive impact on the reduction in oxidative stress [51].

Motor recovery after trauma and quercentin treatment was also investigated in two different models. Thipkaew and colleagues subjected rats with streptozotocin (STZ)-induced diabetes to sciatic nerve crush and administered electrospun nanofiber mats loaded with 5%, 10%, or 15% quercetin to the lesion site [52]. Quercentin treatment resulted in fully recovered sciatic nerve motor function after 21 days, while recovery of the sensory function failed in these diabetic rats.

Huang and colleagues studied the effect of quercitin on oxidative stress and inflammation in a model of C5–C7 nerve root avulsion [53]. After lesion, in situ injections of poly(D,L-lactide-co-glycolide)-poly(ethylene-glycol)-poly(D,L-lactide-co-glycolide)(PLGA-PEG-PLGA) hydrogel, loaded with different concentrations of quercetin, were reported to have promoted axonal outgrowth and recovery of peripheral nerve function through better muscle reinnervation.

Myricetin was isolated for the first time from the bark of the tree Myrica rubra. It is a polyhydroxy flavonol compound with a physical appearance in the form of light-yellow crystals, with a high solubility in methanol, acetonitrile, ethanol, and other polar solvents. It is largely distributed in natural plants, such as Myricaceae, Vitaceae, Leguminosae, Primulaceae, Rosaceae, Ericaceae, Fagaceae, and Compositae, but it is also present in berries, fruits, vegetables, honey, and red wine [54,55,56,57]. An extensive scientific literature report evidences the antioxidative properties of myricetin [58]. Additionally, hepatoprotective, antitumor, anti-inflammatory, analgesic, and antidiabetic properties have been reported [59]. In the central nervous system, myricetin has demonstrated an important role in the promotion of neuronal proliferation in the subgranular and subventricular zone of the hippocampal dentate gyrus [60]; its iron ion chelating properties reduced the brain iron ion content by the inhibition of transferrin receptor 1 expression and significantly reduced the cognitive dysfunction in mice in an Alzheimer’s disease model [61]. To our knowledge, only one scientific study has investigated the role of myricetin in peripheral nerve regeneration after lesion (Table 3). Zhang and colleagues administered myricetin in different doses after rat sciatic nerve crush injury [62]. The authors report significantly increased functional recovery in myricetin-treated rats, as demonstrated for SFI and toe spread index (TSI) values in comparison with control injured animals. Moreover, the increased axonal regeneration and myelination observed in the treated groups was accompanied by enhanced levels of BDNF, an activation of Akt, and subsequently increased levels of p-mTORC1 and p-GSK3β, which are all key players in axonal regeneration signaling cascades [62].

### 3.4. Flavones

Flavones display a chemical structure with two benzene rings and can be found in acerola, apricot, cashew, cabbage, cardon, dandelion, artichoke, mango, papaya, onion, and beans. Among the beneficial effects of these compounds, health-promoting effects for diabetes, depression, Alzheimer’s disease, and cancer are reported [17].

Apigenin is found as a single ingredient in chamomile tea, but it is also a component of red wine and beer and is abundant in a variety of fruits and vegetables (in particular parsley, celery, vine-spinach, artichokes, and oregano) [63,64].

Apigenin has been demonstrated to have several beneficial properties on human health, including antiphlogistic and antibacterial activities. Furthermore, it is reported to be useful in the treatment of asthma, neuralgia, and Parkinson’s disease, as well as to counteract coronary artery disease, gastrointestinal irritation, dermatological disorders, and to provide antidepressant, calming, and relaxing effects. Furthermore, extensive studies have shown apigenin to possess anticancer properties, especially through responses to oxidative-stress-related DNA damage, the inhibition of cancerous angiogenesis and inflammation, suppression of cell growth, and induction of apoptosis and autophagy [64].

With regard to effects on the peripheral nervous system, we found only one study investigating the effect of apigenin on nerve regeneration in vitro. Here, treatment of mouse sciatic nerve ex vivo explants with apigenin solution (0.1, 1 or 10 mM) was reported to inhibit myelin fragmentation in a Krox20-independent manner, and thereby also to inhibit axonal degradation after nerve transection. Moreover, apigenin treatment was reported to inhibit Schwann cell proliferation, and Schwann cell phenotypic change into repairing Schwann cells in an ERK-signaling-independent manner [65].

### 3.5. Isoflavones

Isoflavones are mostly referred to as phytoestrogen, in which the main representative compounds are genistein, daidzein, biochanin A, and glycitein. They are mainly found in legumes, but other sources include apple, apricot, potato, onion, and melon [66].

Isoflavones are polyphenols primarily found in soybean and soybean products, which contain mainly daidzein and genistein. Since isoflavones have a structure and size similar to that of estrogen (in particular to estradiol, 17-β-estradiol, E2), they are often referred to as phytoestrogens and have been shown to exert multiple estrogenic and/or antiestrogenic-related effects [67].

Both genistein and daidzein exhibit a wide range of important properties, including antioxidative and anti-inflammatory activities, for which they are considered chemo-preventive compounds. On the other hand, antiangiogenic, proapoptotic, and antiproliferative activities are considered to have potential. However, again, other beneficial effects have been reported, such as alleviating menopausal symptoms, reducing the incidence of cardiovascular disease, osteoporosis, obesity, diabetes, and cognitive functions [68,69].

Despite the abovementioned wide range of effects exerted by isoflavones, only one study reported the role of genistein on peripheral nerve regeneration after traumatic injury [70] (Table 4). After sciatic nerve crush or end-to-end repair, the authors found better sciatic function index and better paw mechanical withdrawal threshold after four weeks. By analyzing nerve tissue, they also showed higher GAP-43 and myelin basic protein (MBP) immunoreactivity and lower proinflammatory cytokine levels in the regenerated sciatic nerve (IL-1β and TNF-α) compared with untreated animals, and suggested that genistein improved nerve regeneration after traumatic nerve injury and repair [71].

It has been shown that genistein has antioxidant and anti-inflammatory activities on peripheral nerves, because it increased the level of the antioxidative enzymes glutathione peroxidase (GPX) and catalase (CT) and inhibited the expression of proinflammatory cytokines (IL-1b and IL-6) in a model of neuropathic pain [72] and in a diabetic mouse model [73]. Moreover, genistein restored the NGF content in the diabetic sciatic nerve [73]. The administration of daidzein on primary rat DRG neuronal cultures in vitro resulted in neurite outgrowth similar to that induce by NGF treatment, without apparent cell death. To investigate the signaling mechanism underlying the neuritogenic effect of daidzein, the authors tested various inhibitors to DRG neuronal outgrowth before and during daidzein treatment. Both the Src kinase inhibitor and the MEK inhibitor significantly reduced daidzein-induced neuritogenesis. Moreover, Src kinase inhibitor inhibited the daidzein-induced upregulation of protein kinase C delta (PKCδ), ERK1, and ERK2. These data confirmed the involvement of Src kinase, PKCδ, and MEK in the enhancement of daidzein-mediated neurite outgrowth [74].

No data about the effect of daidzein on nerve regeneration in vivo have been found during our literature search.

## 4. Non-Flavonoids

### 4.1. Phenolic Acids: Hydroxybenzoic Derivatives

Hydroxybenzoic acid can be found in certain red fruits, black radish, and onions [75]. Hydroxybenzoic acids are components of complex structures, such as gallotannins (found in mangos) and ellagitannins (found in red fruit such as strawberries, raspberries, and blackberries) [76].

The most common benzoic acid derivatives comprise gallic acid mostly present in tea, mango, rhubarb, soy, tannic acid, protocatechuic acid, and capsaicin.

Protocatechuic acid is widely distributed in nature and is found in common vegetables, fruits, cereals, teas, and some plant drinks. It is further present in some herbal medicines, in buckwheat, mustard, nipa palm nut, kiwi fruit, blackberries, strawberries, chokeberries, and mango. In addition, it is found in chicory, olives, dates, grapes, cauliflower, lentils, onion, garlic, sharp-leaf galangal (Alpinia oxyphylla), and several other plants [71]. It exhibits many different properties, including anti-inflammatory, antioxidant, antiviral, antihepatotoxic, free radical scavenger, antiplatelet aggregation, antidiabetic, antiapoptotic, and neuroprotective properties [71,77].

Regarding the effects on the peripheral nervous system, protocatechuic acid has only been tested in vitro on the RSC96 Schwann cell line, where it enhanced cell survival, proliferation, and migration [78,79]. Indeed, the treatment significantly induced the phosphorylation of the insulin-like growth factor-I (IGF-I)-mediated phosphatidylinositol 3 kinase/serine–threonine kinase (PI3K/Akt) pathway [78], modulated apoptosis by suppressing Bad (a proapoptotic protein), upregulating the pro-survival proteins p-Bad and Bcl-xL, and increasing the expression of the proliferating cell nuclear antigen (PCNA). G1–S-phase cell cycle transition also increased, as demonstrated by an increase in the expression of the cell cycle regulators cyclins D1, E, and A [78]. Moreover, the phosphorylation of MEK1/2, ERK1/2, JNK1/2, and p38 increased after protocatechuic acid treatment, leading to the expression of proteolytic enzymes of tissue type (tPA) and urokinase type (uPA). Finally, zymography results demonstrated that the activity of metallopeptidase 9 (MMP9) and MMP2 was increased after treatment of RSC96 Schwann cells with protocatechuic acid [79].

Gallic acid is found with high content in tea, grapes, and wine [80]. It has reported antioxidant, antihyperglycemic, antihyperlipidemic, anti-inflammatory, anticancer, and neuroprotective effects [81,82], even against age-related neurodegenerative disorders, including Parkinson’s disease, amyotrophic lateral sclerosis, and Alzheimer’s disease [83]. It has also been shown that gallic acid reverses cyclophosphamide- and paclitaxel-induced neurotoxicity [84] and that it has antinociceptive and antiedematogenic effects in a chronic constriction injury neuropathic pain mice model [85].

Table 5 summarizes methodological details of the two in vivo studies that focused on the effects of gallic acid on peripheral nerve regeneration, while study results are described in more detail in the following text.

Oral administration of gallic acid showed a dose-dependent improvement of motor function, coordination, and nerve conduction velocity, but not of the pain sense reflex after rat sciatic nerve crush injury [86]. However, no data about axon regeneration or remyelination were presented by the authors.

After a more severe injury (end-to-end repair of the rat sciatic nerves), systemic administration of gallic acid resulted in reports of improved motor function (higher degrees climbed in the inclined plane test) and electrophysiological results (greater amplitudes of CMAP and reduced CMAP latency) compared with the saline-treated animals. Histologically, regenerated sciatic nerve presented more myelinated fibers, less fibrosis, and increased NGF immunoexpression in the Schwann cells. Moreover, animals treated with gallic acid also displayed a decrease in the oxidative stress (lower lipid peroxidation) and lower inflammation (higher heat shock protein-70, HSP-70 values), representing the anti-inflammatory and antioxidant effects of gallic acid [87].

Ellagic acid is a polyphenol compound abundantly present in berries (strawberry, raspberry, cloudberry, and blueberry), grapes, pomegranate, almonds, walnuts, and beverages [88]. It possesses broad-spectrum physiological activities including antioxidative, anti-inflammatory, antibacterial, anticarcinogenic, antiplasmodial, antiviral, hepatoprotective, antifibrotic, and immunomodulatory activities. It exerts also appreciable neuroprotective activity by its free-radical-scavenging action, iron chelation, initiation of several cell signaling pathways, and alleviation of mitochondrial dysfunction [89].

A positive effect of ellagic acid has been shown in tibial and sural-nerve-transection-induced neuropathic pain [90]. Oral administration of pomegranate fruit extract (containing 41.6% ellagic acid, 10% Punicalagins, 5.1% Granatin) was reported to have anti-inflammatory properties, nitric oxide inhibition capacity, and the ability to decrease oxidative stress [90]. Moreover, ellagic acid has been demonstrated to decrease oxidative stress in sciatic nerves of STZ-induced diabetic rats (reduction in the oxidative stress markers such as MDA, TOS, OSI, and NO) [91]. Since neither study evaluated any specific effects of ellagic acid on peripheral nerve regeneration, we have not provided further details of them on a specific table. The same applies to a recent study that developed a chitosan- and collagen-based scaffold loaded with ellagic acid with the aim of incorporating antioxidative properties within the supporting matrix [92]. The presence of ellagic acid within the scaffold and its biocompatibility was confirmed, and the recovery of primate kidney fibroblasts from UV-irradiation-derived oxidative stress was demonstrated [92]. The authors proposed that this scaffold could support wound healing. However, collagen and chitosan are natural polymers extensively used for tissue engineering applications and have demonstrated supporting properties also in the field of peripheral nerve regeneration [93,94]. Therefore, the approach could be interesting for providing local administration of ellagic acid (as well as other antioxidant compounds) during the process of nerve regeneration.

### 4.2. Phenolic Acids: Hydroxycinnamic Acids

p-Hydroxycinnamic acids (p-coumaric, ferulic, sinapic, and caffeic acids) are phenolic compounds involved in biosynthesis of lignin [95]. They have been known for centuries for their multiple biological and therapeutic properties, such as their anticancer, antidiabetic, and anti-inflammatory activities [96], and may prevent thrombosis and neurodegenerative diseases [97,98].

Hydroxycinnamic acids can be found in derivative forms, such as amides (combination with amino acids or peptides) and esters (combination with hydroxyl acids or glycosides).

p-Coumaric acid is the major precursor in the synthesis of other phenolic acids, such as caffeic, chlorogenic, rosmarinic, and ferulic acids. It is widely distributed in fruits, vegetables, cereals, and mushrooms, and plays a role as an antioxidant, antimicrobial, antitumor, anti-inflammatory, and antiplatelet aggregation factor [99].

Among p-Coumaric derivatives, curcumin is a natural compound extracted from the roots of Curcuma longa that exhibits a variety of pharmacologic properties, including anti-inflammatory, anticarcinogenic, and antioxidant activities. Furthermore, it has been increasingly recognized as a neuroprotective molecule both in the central and peripheral nervous system.

Many different in vivo studies focused on the effects of curcumin on peripheral nerve regeneration. Table 6 summarizes details on peripheral nerve lesion model, administration roots, and experimental groups for in vivo studies on curcumin and ferulic acid. The respective study results are described in the following paragraphs.

Systemic administration of curcumin showed a dose-dependent effect in promoting nerve regeneration after crush injury with regard to increased numbers of retrogradely labeled regenerating motoneurons, myelinated axons, and bigger nerve fiber diameters compared with untreated rats [100,101,102]. Increased remyelination was related to elevated expression of S100, peripheral myelin protein 22 (PMP22), MBP, and MAG, as well as to a decrease in fibrin deposition [103]. Moreover, motor functional recovery, CMAP latency of onset, peak amplitude of CMAP, and NCV were better after high doses of curcumin administration, with a partial reversion of gastrocnemius muscle atrophy [100]. The same functional result was also observed in a mouse model of end-to-end repair of the sciatic nerve, where intragastric administration of curcumin at high doses increased the action potential amplitude and the MNCV [101].

Systemic, intraperitoneal administration of curcumin daily (100 mg/kg) for 4 weeks after rat sciatic nerve end-to-end repair resulted in increased sciatic nerve functional index (SFI) values for rats additionally treated with a chitosan membrane around the suture site. Furthermore, morphometrical analysis of regenerated fibers displayed a higher number of regenerated myelinated fibers and increased fiber diameters and myelin thickness [104]. Although the mechanism of curcumin’s neural protection is complex and still needs to be clarified, Sang and colleagues reported the possibility that curcumin exerts a neuroprotective effect via regulation of PI3K/Akt and NGF secretion, reducing the number of apoptotic cells through upregulation of TrkA and Akt and downregulation of p17 [105]. In the same study, the antioxidant effect of curcumin was compared to methylprednisolone and propolis, displaying no significant differences in terms of myelin thickness, axon diameter, nerve diameter, or *g*-ratio among the three groups [105]. Treatment with curcumin also reduced the production of reactive oxygen species (ROS) and lipid peroxidation and increased expression of transcription factor Nrf2 [106].

The effect of curcumin was also investigated regarding autophagy, myelination, and related signal transduction pathways, Erk1/2 and Akt, by Zhao et al. [103]. They demonstrated that the promoting effect of curcumin on remyelination and axon regeneration was reversed by an Erk1/2 pathway inhibitor and Akt pathway agonist during the recovery of injured sciatic nerves after crush lesion [103].

In a different experimental setting, the effect of curcumin was compared with that of melatonin, a neuroprotective, antioxidant, and anti-inflammatory drug, known to be affected by the circadian rest–activity cycle. The authors report that curcumin displayed better circadian-rhythm-independent performance than melatonin in stimulating nerve regeneration [107].

Another in vivo study tested a hollow poly-L-lactic acid (PLLA) tube filled with fibrin hydrogel containing Schwann cells or curcumin encapsulated within chitosan nanoparticles for repairing a 10 mm rat sciatic nerve gap. The subsequent release of curcumin from its encapsulation over time demonstrated significant support of sciatic nerve regeneration. As revealed by histological evaluation, curcumin had a specific effect on Schwann cells. Furthermore, motor and nociceptive functional recovery displayed no significant difference among the experimental groups, indicating that curcumin-containing conduits performed comparably to nerve autografts [108].

The possible local effect of curcumin was also investigated by administering curcumin within a silicone tube used to repair a 10 mm gap sciatic nerve injury. Functional recovery was significantly improved, and a morphometrical analysis displayed a significant increase in regenerated fibers and fiber size parameters in curcumin-treated rats compared with the control group [109].

Ferulic acid is a component of Angelica sinensis Diels and Ligusticum chuanxiong Hort, with free radical scavenger, anti-inflammatory, and antioxidation properties. Although the literature shows that ferulic acid has versatile biological functions, its nerve-growth-promoting effect has been poorly characterized (see Table 6 for details on model, administration, and experimental groups).

Intraperitoneal administration of ferulic acid after sciatic nerve crush resulted in a 1-fold increase in MAG expression and a 2-fold increase in MBP expression compared with the expression levels in the vehicle-treated control group. The authors further reported on higher proliferation of primary Schwann cells after ferulic acid treatment versus control [110].

The effects of ferulic acid have been investigated in vivo in a model of silicone rubber tube filled with ferulic acid used to repair a 15 mm rat sciatic nerve defect [111]. The authors report a beneficial effect of ferulic acid in axonal regrowth, highlighting the presence of myelinated axons and regenerated nerve cables within the bridging conduits, and a significantly shortened latency and accelerated nerve conduction velocity (NCV) of the evoked CMAPs in comparison with the vehicle-treated control condition [111].

### 4.3. Lignans

Lignans are another kind of non-flavonoid polyphenols that can, similarly to the isoflavones, bind to estrogen receptors and function as phytoestrogens. They are bioactive compounds exhibiting various biological antitumor, antioxidant, and anti-inflammatory activities. Flax seeds and sesame seeds contain high levels of lignans, but lignans are found in relatively low concentrations in other foods, including cereals (rye, wheat, oat, and barley), soybeans, vegetables, such as broccoli and cabbage, and some fruits (apricots and strawberries) [112].

No studies are present in the literature investigating the effect of isolated compounds on peripheral nerve regeneration; however, two studies have investigated the effect of flaxseed oil and sesame oil, both rich in lignans (see Table 7 for details on model, administration, and experimental groups).

Flaxseed oil contains high levels of lignans, but it also has the richest source of α-linolenic acid, the ω-3 polyunsaturated fatty acid (PUFA), which is an essential fatty acid for humans. Rats orally treated with flaxseed oil showed earlier functional recovery after sciatic nerve crush injury compared with untreated animals, and regenerated myelinated nerve fibers presented increased myelin thickness [113].

Additionally, sesame oil contains high percentage of lignans (sesamin, sesamolin, sesamol), together with fatty acids (palmitic acid, palmitoleic acid, stearic acid, oleic acid, linoleic acid, linolenic acid, and eicosenoic acid) and other antioxidants (α-tocopherol). Sesame oil has been reported to possess excellent antioxidative and anti-inflammatory properties and has been demonstrated to improve nerve functional recovery after rat sciatic nerve crush injury by attenuating nerve oxidative stress [114]. Indeed, treatment with sesame oil significantly decreased lipid peroxidation levels (a biomarker of oxidative stress) in serum and sciatic nerve tissue and increased the antioxidant nuclear Nrf2 expression [114].

### 4.4. Stilbenes

Stilbenes are a class of plant polyphenols that have attracted intense interest for their intricate structures and biological activities. These compounds do not have a wide distribution in the plant kingdom, and phytochemical investigation is mainly focused on a few families [115].

Stilbenes have received extensive attention for their various functions in a healthy human diet and medical treatments, such as their antioxidative and anticancer activities. This class of compounds has shown extraordinary potential in the biomedical fields, especially in the treatment of neuroinflammation [116].

Stilbenes are present in grapes, bean, almond, bilberries, blueberries, peanuts, grapevine, cranberries, and wine; these molecules present variable structure with diversity in chemical functional groups and polymerization that determine their absorption and metabolism rate. Among the stilbenes, the most studied is resveratrol, which exerts a role in reducing body weight and in cardiovascular system protection [17]. Stilbenes classification divides them into five groups according to the constituent units [115]. Resveratrol has also been described with regard to its benefits in controlling multiple targets involved in oxidative stress, inflammation, cell death, and immune response [117].

Regarding the role of resveratrol in peripheral nerve injury and regeneration, an in vivo study showed that the motor deficits caused by sciatic nerve crush injury were alleviated by daily systemic resveratrol treatment within 10 days [118] (see Table 8 for details on model, administration, and experimental groups).

Starting from the neuroprotective effect of resveratrol, the authors hypothesized that it could upregulate the expression of VEGF and thereby mediate nerve regeneration and motor repair. Functional analysis displayed that the SFI was increased by the resveratrol administration and morphological analysis of regenerated nerve fibers indicated a better axonal regeneration than in vehicle-treated control rats. The latter was supported by immunofluorescence analysis for axonal markers displaying more NF200-positive axons in resveratrol-treated rats [118].

Another in vivo study investigated the effect of resveratrol in a rat model of sciatic nerve chronic constriction injury by behavioral, histomorphological, and immunohistochemical analysis [119]. Although it is well known that this type of lesion is more suitable for pain evaluation, we provide further details of this study on the table since authors focused on nerve regeneration. Indeed, morphometrical analysis revealed that resveratrol treatment increased axon numbers and supported myelin morphology towards almost comparable conditions to healthy nerve fibers in contrast to saline treatment. Resveratrol treatment restored the IGF-1 expression that was reduced in the saline-treated group and prevented motor impairment caused by injury increasing the walking distance and total entries in the open field test [119].

A novel role attributed to resveratrol is the promotion of autophagy that occurs extensively in a wide variety of cell types. In the peripheral nervous system, Schwann cells play a key role in the recovery of injured nerves not only by secreting neurotrophic factors, but by autophagy for myelin removal after injury. Indeed, after injury, degradation of myelin sheath occurs distal to the lesion site and is dependent not only on phagocytosis by macrophages but also on autophagy by Schwann cells during Wallerian degeneration [120,121,122]. The effects of resveratrol on autophagy were assessed in vitro on the RSC96 Schwann cell line, in which resveratrol significantly increased autophagy, as revealed by the by the colocalization of the autophagy markers LC3B and p 62 [123].

Autophagy induction was also evaluated after sciatic nerve crush injury in rats, where treatment with resveratrol resulted in the presence of autophagic bilayer membrane structures and typical autophagic fragments of the enclosed myelin sheaths, detectable with transmission electron microscopy. Immunofluorescence analysis confirmed the presence of autophagy displaying a colocalization of myelin protein zero and LC3B. Furthermore, double labeling of S-100β and LC3B showed autophagy had high occurrence in Schwann cells [123].

The effect of resveratrol was also described to be evident in changes in the composition and condition of lipids within the peripheral nerve after transection injury. Lipid extraction, from nerves that underwent electrical stimulation or have been injured, was carried out to estimate their content of phospholipids (PL), phosphatidylethanolamine (PEA), diacylglycerol (DAG), fatty acid contents (FA) and free fatty acids (FFA) (particularly important during hydrolysis). Animals injected with resveratrol displayed an increased lipid content of PEA, phosphatidylcholine (PCH), and sphingomyelin (SM) in the proximal part of the nerve. In the distal segment the PS content was reduced, and the DAG level increased compared with untreated injured nerves. The stabilizing effect of resveratrol on the phospholipid composition of nerve fibers could be explained by the fact that the resveratrol molecule is lipophilic and can be transported and facilitate the penetration of large amounts of molecules through cellular membranes. One of the mechanisms of action of resveratrol on the nerve regeneration process is associated with its antioxidant ability, due to the leveling of the content of hydroxyl, superoxide, and other radicals. It has been speculated that changes in the content of PL and DAG, as well as the redistribution of fatty acids of various lipid fractions, have an effect on the protein lipid relationships and the orderliness of fatty acids (viscosity) in the myelin and axolemma of the nerve fiber [124].

### 4.5. Tannins

Tannins (commonly referred to tannic acid) are water-soluble, non-flavonoid polyphenols present in many plant-derived foods [125]. They have been reported to be responsible for a decrease in food intake, growth rate, feed efficiency, net metabolizable energy, and protein digestibility in experimental animals. Therefore, foods rich in tannins are considered to be of low nutritional value.

Tannins are constituents of beans, nuts, and berries, and are mainly divided into hydrolyzable and non-hydrolyzable forms that exert important properties in the prevention of urinary tract infections, as well as in the prevention of a wide range of chronic conditions [17].

Regarding peripheral nerve regeneration, it has been proven that tannic acid exhibits anticorrosive properties in metal-based devices. We found only one study in which an in vitro analysis was performed on primary Schwann cells cultured in presence of Mg–Ca alloy ribbons with a coating of tannic acid. The presence of tannic acid in the device was reported to increase its biocompatibility, as demonstrated by the MTT assay and the analysis of the cell morphology. The authors concluded that Mg-based metallic glasses in the Mg-Zn-Ca system may represent a promising biomaterial with potential for nerve regeneration and as implantable nervous prosthetic devices [126]; although, the in vivo evidence is outstanding.

## 5. Discussion

A balanced diet is necessary for maintaining a healthy body, since it supplies the nutrients our bodies need to work effectively. In recent decades, there has been increasing interest in investigating the impact of dietary nutrients on peripheral nerve regeneration [6,127]. We recently presented a review about the role of macro- and micronutrient supplementation, as well as different dietary regimens, in improving peripheral nerve regeneration [8]. The present review adds to this by summarizing the current knowledge on the effects of mainly food-derived polyphenolic compounds on the process of nerve regeneration after traumatic injury.

The role of dietary polyphenols has gained growing importance in food and nutrition research, as demonstrated by the increased number of publications over the past 20 years. This increasing interest in polyphenol research is probably due to the fact that they are a large family of hundreds of secondary metabolites with diverse structures that can be found largely in fruits, vegetables, legumes, cereals, and plant-derived beverages, i.e., juices, red wine, tea, and coffee, and are consumed daily through different food products and preparations. The biological activity of polyphenols has been long investigated using a variety of in vitro and in vivo experimental models that have attributed a wide range of properties to these compounds, contributing to maintaining health and to preventing, delaying, or reducing symptoms of some chronic diseases [10]. Polyphenols have an emerging clinical utilization as antioxidants acting as metal chelators, antimutagens, anticarcinogens, and antimicrobial agents in many diseases [11,12]. Phenolic acids were further reported to have neuroprotective effects in the nervous system, ameliorating depression, ischemia/reperfusion injury, neuroinflammation, neuropathic pain [13], glutamate-induced toxicity, epilepsy, hearing and vision disturbances, and many other neurodegenerative diseases [14].

**Figure 3:**
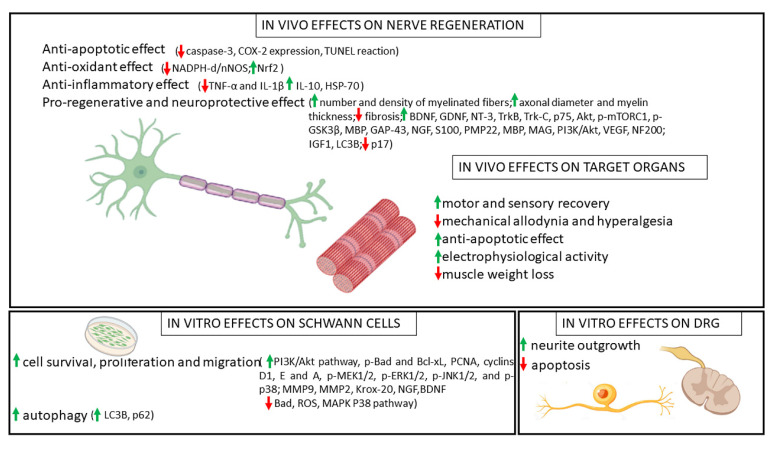
Beneficial effects of polyphenols on nerve regeneration with details of molecular mechanisms.

As mentioned above, many studies, both in vitro and in vivo, have highlighted the beneficial effects of polyphenols in regenerating the peripheral nervous system by evaluating motor and sensory functional recovery. These effects were linked to the reduction in (neuro-)inflammation, a support of axonal regeneration, and the improved morphology of regenerated nerves. Most of the positive effects of polyphenols have further been associated with their anti-inflammatory, antiapoptotic, and antioxidative properties. Oxidative stress is indeed one of the main factors of neural damage after injury; therefore, the reduction in injury-triggered inflammatory response and the inhibition of oxidative stress could positively support the regenerative process after nerve injury [128]. Figure 3 summarizes the molecular mechanisms associated with the beneficial effects of polyphenols on nerve regeneration and target organ recovery in vivo, together with the main findings derived from in vitro studies on Schwann cells and dorsal root ganglion sensory neurons.

However, it is noteworthy that most of the in vivo studies describing such positive effects of polyphenols on the regenerating peripheral nervous system used the rat sciatic nerve crush injury model (axonotmesis) for investigation. Crush injury leads to axonal damage without physical disruption of connective tissue sheaths and is therefore characterized by a spontaneous and fast regeneration. A more common and challenging clinical problem involves, however, more severe nerve injuries; in the worst cases, these are complete nerve transection injuries (neurotmesis) with substance loss. Therefore, future studies are needed for assessing whether these compounds could also have a positive effect under more challenging conditions. The design of any future studies should, in the opinion of the authors, also consider the use of well-standardized and unbiased evaluation methods for functional [129] and histological [130,131] evaluation.

From the literature review provided above, only two groups of polyphenols—flavonols, in particular quercetin [48], and hydroxycinnamic acids, in particular curcumin [108,109] and ferulic acid [111]—can be confirmed to have a supporting effect on regeneration after nerve gap repair. For most other polyphenol compounds, less knowledge exists on their effect on regenerating the peripheral nervous system, and knowledge derived from in vitro studies has not yet been further investigated in vivo.

The reason for this is most likely related to the hydrophobic property, low solubility, and minimal bioavailability of polyphenols for clinical applications. The bioavailability of polyphenols especially varies among the different classes, and it has recently been ranked from their experimental use in vivo, as follows: phenolic acids > isoflavones > flavonols > catechins > flavanones, proanthocyanidins > anthocyanins [132]. The low bioavailability could be attributed to the interaction with the food matrix components on one side, or to the metabolic processes mediated by the liver, intestine, and gut microbiota on the other side. The lipophilic property per se does not oppose the clinical use of the respective compounds, because lipophilic substances could exert neuroprotective function thanks to their ability to penetrate within cellular membranes [124]. Moreover, alternative strategies for increasing the in vivo availability of polyphenols through their delivery via, e.g., encapsulation into nanocarriers or similar strategies, could increase their experimental and subsequent clinical use for supporting peripheral nerve regeneration and preventing secondary impairment after peripheral nerve injury, such as neuropathic pain. Despite this beneficial outlook, future studies are needed for ensuring that the negative side effects will not develop from systemic administration of polyphenol-derived compounds during the course of recovery after peripheral nerve trauma. As an example, there is—largely based on their estrogen-like properties—a growing concern for the safety of soyfoods, isoflavones, and specific non-flavonoids in flaxseed or sesame oil, in spite of the considerable enthusiasm regarding their potential health benefits.

To date, there are no active clinical trials investigating the effect of polyphenols on damaged peripheral nerves of humans. A recent review summarized all the registered clinical trials on polyphenol effects on human health, disease (i.e., cancer, urogenital diseases, and psychiatric disorders), and on subjects with risk factors (i.e., high blood pressure, fasting glucose, and triglyceride levels) in the last two decades (2000–2020). A total of 750 clinical trials have been included in the review, highlighting the need to effectively investigate the role of polyphenols activity on human health and disease, while considering the inter-individual variability in response to the intake of polyphenols that could affect polyphenols bioavailability and bioactivity [133].

**Figure 4:**
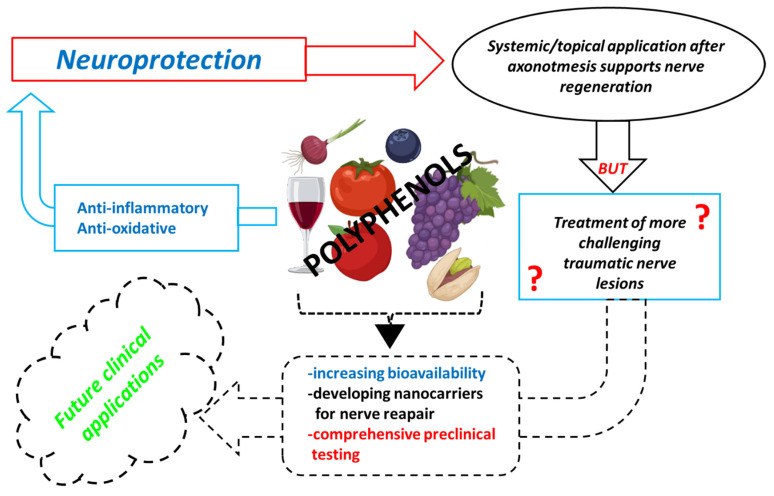
Schematic representation of the polyphenols neuroprotective effects described in the literature: limits regarding administration and choice of experimental models and new perspectives.

In summary, here we provide a literature review proposing the potential beneficial activities of polyphenols on the processes of peripheral nerve regeneration. In vivo and in vitro studies showed anti-inflammatory, antiapoptotic, antioxidative, and pro-regenerative properties, with beneficial effects for target organs also. However, new strategies for increasing their bioavailability or for optimizing the possibilities of their secure local delivery are warranted. Future studies are needed for a deeper understanding of the biomolecular mechanisms underlying their activity, of their biological safety when administered as therapeutic compounds, and especially of the effect of polyphenols on more severe peripheral nerve injuries. Finally, future clinical trials to demonstrate the benefits of polyphenols for humans should be conducted (Figure 4).

## Figures and Tables

**Table 1:** Flavanols: Table showing the experimental method (type of nerve injury and animal model), the dose, and method of administration of epigallocatechin gallate (EGCG) and green tea extract (GTP). Included references are listed ascending in accordance to their publication date.

Epigallocatechin Gallate		
Ref.	Type of Nerve Lesion and Animal Model	Type of Administration/Experimental Groups
Kian et al., 2019 [29]	Sciatic nerve transection; male Sprague Dawley rats	- Sham-operated rats; - Sciatic nerve transaction with saline (vehicle); - 50 mg/kg of EGCG intraperitoneally 30 min before nerve transection and followed for 3 days; - 50 mg/kg of EGCG intraperitoneally 1 h after nerve transection and followed for 3 days.
Renno et al., 2017 [26]	Sciatic nerve crush injury; male Wistar rats	- Control (no injury); - Sham-operated rats; - Crush + saline-treated rats; - Crush + 50 mg/kg; - Intraperitoneal injection of EGCG or saline daily for 3 days starting 1 h after nerve injury.
Renno et al., 2016 [28]	Sciatic nerve crush injury; male Wistar rats	- Control (no injury); - Sham-operated rats; - Crush + saline-treated rats; - Crush + 50 mg/kg EGCG; - Intraperitoneal injection of EGCG or saline daily starting 1 h after nerve injury until sacrifice.
Yildirim et al., 2014 [27]	Sciatic nerve crush injury; male Albino Wistar rats	- Control (no injury); - Injury without treatment; - Intraperitoneal injection of saline for 7 days; - Intraperitoneal injection of 25 mg/kg EGCG for 7 days; - Intraperitoneal injection of 50 mg/kg EGCG for 7 days; - Daily consumption group (intraperitoneal injection of 10 mg/kg EGCG for 14 days before injury). Samples were harvested at 28 days after injury.
Renno et al., 2013 [25]	Sciatic nerve crush injury; male Wistar rats	- Sham-operated rats; - Crush + saline-treated; - Crush + 50 mg/kg EGCG. Intraperitoneal injection of EGCG or saline daily for 3 days starting 1 h after nerve injury.
Renno et al., 2012 [24]	Sciatic nerve crush injury; male Wistar rats	- Sham-operated rats; - crush + saline-treated; - crush + 50 mg/kg EGCG; Intraperitoneal injection of EGCG or saline 1 h after nerve injury, and days 1 and 2 post-surgery.
Wei et al., 2011 [30]	Left vagus and hypoglossal nerve crush injury; male Wistar rats	- Sham-operated rats; - Injury without treatment; - 10 mg/kg EGCG pretreatment; - 25 mg/kg EGCG pretreatment; - 50 mg/kg EGCG pretreatment. Daily intraperitoneal injections of EGCG for successive six days with the last injection at 30 min before injury.
**Green tea extract**		
Chen et al., 2020 [37]	Sciatic nerve end-to-end repair; male Wistar rats	- Vehicle group: intraperitoneally injected with saline for 2 weeks; - Green tea group: intraperitoneally injected with GTPs (50 mg/kg/d) for 2 weeks.
Zhou et al., 2015 [36]	10 mm-long sciatic nerve defect repaired with allograft; male Wistar rats	- Autograft; - Fresh nerve allograft; - Irradiation-pretreated nerve allograft (26.39 Gy/min for 12 h); - Green tea polyphenol-pretreated nerve allograft: nerve segments immersed in DMEM solution containing polyphenol (1 mg/mL) for 1 week and in DMEM solution for a subsequent 3 weeks at 4 °C.
Nakayama et al., 2010 [35]	30 mm-long ulnar nerve defect repaired with allograft; male and female beagle dog	Nerve fascicles from male dog stored in DMEM containing polyphenol (1 mg/mL) for one week and then transferred to DMEM solution alone for three weeks. These nerve segments were used to repair the right female ulnar nerves. The left ones were repaired with autograft.After nerve repair, the immunosuppressant FK506 administration was started one day before the transplantation, at different doses: - Subcutaneous injections of 0.1 mg/kg FK506 every day; - Subcutaneous injections of 0.05 mg/kg FK506 every day; - Subcutaneous injections of 0.05 mg/kg FK506 every other day.
Ikeguchi et al., 2005 [34]	15 mm-long sciatic nerve defect repaired with allograft; inbred Lewis rats and male Dark Agouti rats	- Isograft group: nerve segments harvested from male Lewis rats and immediately transplanted into male Lewis rats. - Polyphenol-treated isograft group: nerve segments harvested from male Lewis rats. - Stored in DMEM containing polyphenol (1 mg/mL) for 4 weeks, and then in DMEM solution alone for 2 days and transplanted into male Lewis rats; - Fresh allograft group: nerve segments harvested from male DA rats and immediately transplanted into male Lewis rats.
Matsumoto et al., 2005 [33]	15 mm-long sciatic nerve defect repaired with allograft; inbred Lewis rats	- Fresh nerve graft group (immediate repair with nerve segment). - Nerve deficit group (no repair). - Nerve segments used to repair the nerve gap were treated with different concentrations of polyphenols for different periods of immersion: - 1.0 mg/mL polyphenol for 1 day and then in DMEM for 27 days at 4 °C; - 1.0 mg/mL polyphenol for 1 week and then in DMEM for 3 weeks at 4 °C; - 1.0 mg/mL polyphenol for 4 weeks and then in DMEM for 2 days at 4 °C; - 0.5 mg/mL polyphenol for 1 day and then in DMEM for 27 days at 4 °C; - 0.5 mg/mL polyphenol for 1 week and then in DMEM for 3 weeks at 4 °C; - 0.5 mg/mL polyphenol for 4 weeks and then in DMEM for 2 days at 4 °C; - 2.5 mg/mL polyphenol for 1 day and then in DMEM for 27 days at 4 °C; - 2.5 mg/mL polyphenol for 1 week and then in DMEM for 3 weeks at 4 °C; - 2.5 mg/mL polyphenol for 4 weeks and then in DMEM for 2 days at 4 °C.
Ikeguchi et al., 2003 [32]	15 mm-long sciatic nerve defect repaired with allograft; male (donor) and female (recipient) Lewis rats	- Nerve segments removed and transplanted without any storage; - Nerve segments immersed in DMEM solution containing polyphenol (1 mg/mL) for 1 week and in DMEM solution for a subsequent 3 weeks at 4 °C; - Nerve segments immersed in DMEM solution for 4 weeks at 4 °C.

**Table 2:** Flavanones: Table showing the experimental method (type of nerve injury and animal model), the dose, and the method of administration of hesperidin (Hes) and naringenin. Alg/Chit—Alginate/Chitosan. Included references are listed ascending in accordance to their publication date.

Hesperidin		
Ref.	Type of Nerve Lesion and Animal Model	Type of Administration/Experimental Groups
Bagher et al., 2020 [38]	Sciatic nerve crush injury; adult male Wistar rats	- Negative control (injury but no surgical interventions); - Positive control (no injury); - Alg/Chit hydrogel; - Alg/Chit/0.1%Hes; - Alg/Chit/1%Hes; - Alg/Chit/10%Hes; Hesperidin (0.1%, 1%, and 10% (*w*/*v*)) was loaded into cross-linked alginate/chitosan hydrogel. The hydrogel was injected to the site of the crush injury.
**Naringenin**		
Ebrahimi et al., 2020 [42]	Sciatic nerve crush injury; male adult Wistar rats	- Negative control (injury without treatment); - Positive control (rats without injury); - Treatment with Alg/Chit hydrogel; - Treatment with Alg/Chit hydrogel containing Berberine (Ber)-loaded Cs; - Nanoparticles (NPs); - Treatment with Alg/Chit hydrogel containing Naringin (Nar)-loaded Cs NPs; - Treatment with Alg/Chit hydrogel containing both Ber and Nar NPs. The hydrogel was injected into the site of crush injury.
Oliveira et al., 2020 [43]	Sciatic nerve crush injury; male Swiss mice	- Sham group; - Vehicle (saline + tween 0.4% *v*/*v*); - Naringenin (50 mg/kg); - Naringenin complexed with hydroxypropyl-β-cyclodextrin (50 mg/kg). Treatment started 24 h after surgery, by oral gavage, for 28 consecutive days.
Samadian et al., 2019 [41]	Sciatic nerve crush injury; male Wistar rats	- Collagen type I hydrogel with naringin; - Collagen type I hydrogel without naringin; - Negative control (injury without treatment); - Autograft group (positive control). The hydrogel was injected into the site of crush injury.

**Table 3:** Flavonols: Table showing the experimental method (type of nerve injury and animal model), the dose, and method of administration of quercetin and myricetin. PLGA-PEG-PLGA—poly (D,L-lactide-co-glycolide)-poly(ethylene-glycol)-poly(D,L-lactide-co-glycolide) hydrogel. Included references are listed ascending in accordance to their publication date.

Quercetin		
Ref.	Type of Nerve Lesion and Animal Model	Type of Administration/Experimental Groups
Chen et al., 2017 [49]	Sciatic nerve crush injury; male C57BL/6J mice	- Quercetin 0.2 mg·kg-1·day-1 (low dose) - Quercetin 2 mg·kg-1·day-1 (medium dose) - Quercetin 20 mg·kg-1·day-1 (high dose) - mNGF 4.86 μg·kg-1·day-1 - saline (vehicle group) - sham group Injection in the plantar muscle on the left hindlimb once daily with a volume of 0.1 ml, starting after nerve injury and for 7, 14 or 35 days.
Huang et al., 2020 [53]	The C5–C7 nerve roots avulsion; C6 anterior root resection and positioning of the posterior root above the anterior root; female Sprague Dawley rats	- Control; - Hydrogel; - 5 mg/mL quercetin-loaded hydrogel; - 10 mg/mL quercetin-loaded hydrogel; - 50 mg/mL quercetin-loaded hydrogel; - 100 mg/mL quercetin-loaded hydrogel. In situ injections of PLGA-PEG-PLGA hydrogel loaded with different concentrations of quercetin.
Qiu et al., 2019 [50]	Sciatic nerve crush; male ICR mice	- Sham-operated group; - Sciatic nerve crush + intraperitoneal injection of saline; - Sciatic nerve crush + intraperitoneal injection of isoquercitrin (20 mg/kg/day).
Turedi et al., 2018 [51]	sciatic nerve crush; Sprague Dawley rats	- Sham-operated group; - Quercetin group (200 mg/kg); - Crush injury; - Crush injury + quercetin (200 mg/kg). Quercetin (200 mg/kg) was administered intragastrically for 7 days or 28 days.
Thipkaew et al., 2017 [52]	Sciatic nerve crush; Wistar male rats with streptozotocin (STZ)-induced diabetes	- DM + crush injury and no treatment; - DM + crush injury + zein-based nanofiber mats treatment; - DM + crush injury + zein-based nanofiber loaded with 5% quercetin; - DM + crush injury + zein-based nanofiber loaded with 10% quercetin; - DM+ crush injury + zein-based nanofiber loaded with 15% quercetin.
Wang et al., 2011 [48]	15 mm rat sciatic nerves gap; adult Sprague Dawley rats	- Silicon rubber chamber filled with saline; - Silicon rubber chamber filled with 0.1 µg/mL QC solution; - Silicon rubber chamber filled with 1 µg/mL QC solution; - Silicon rubber chamber filled with 10 µg/mL QC solution.
**Myricetin**		
Zhang et al., 2018 [62]	Sciatic nerve crush injury; Sprague Dawley rats	- Saline group; - Nerve injury, treated with equivalent volumes of saline; - Nerve injury + myricetin 25 mg/Kg; - Nerve injury + myricetin 50 mg/Kg; - Nerve injury + myricetin 100 mg/Kg. Treatment groups received Myricetin via oral gavage every day for 14 days.

**Table 4:** Isoflavones: Table showing the experimental method (type of nerve injury and animal model), the dose, and method of administration of genistein.

Genistein		
Ref.	Type of Nerve Lesion and Animal Model	Type of Administration/Experimental Groups
Ozbek et al., 2017 [70]	Sciatic nerve crush injury or end-to-end repair; male Sprague Dawley rats	- Sham (no injury); - Control (crush injury); - Crush injury + 20 mg/kg genistein; - Crush injury + 90 mg/kg gabapentin; - End-to-end repair + 20 mg/kg genistein; - End-to-end repair + 90 mg/kg gabapentin. Genistein and gabapentin (control) were administered intraperitoneally for 30 days.

**Table 5:** Hydroxybenzoic derivatives: Table showing the experimental method (type of nerve injury and animal model), the dose, and method of administration of gallic acid. Included references are listed ascending in accordance to their publication date.

Gallic Acid		
Ref.	Type of Nerve Lesion and Animal Model	Type of Administration/Experimental Groups
Gurkan et al., 2021 [87]	Sciatic nerve end-to-end repair; male albino Sprague Dawley	- Control (no injury); - Surgery + saline (1 mL/kg/day 0.9% NaCl); - Surgery + gallic acid (20 mg/kg/day). Gallic acid was administered intraperitoneally following the surgery, for 12 weeks.
Hajimoradi et al., 2014 [86]	Sciatic nerve crush; male Wistar rats	- Control: intact rats received normal saline (2 mL/kg); - Crush + saline (2 mL/kg); - Crush + 50 mg/kg/2 mL gallic acid (GA); - Crush + 100 mg/kg/2 mL GA; - Crush + 200 mg/kg/2 mL GA; - Crush + forced exercise; - Crush + forced exercise combined with 200 mg/kg/2 mL GA as an effective dose for 21 days. Gallic acid was administered orally (dissolved in 2 mL/1000 g body weight normal saline) at different doses every day starting from the 2nd day after nerve crush for 21 days, alone and in combination with exercise.

**Table 6:** Hydroxycinnamic acids: Table showing the experimental method (type of nerve injury and animal model), the dose, and method of administration of curcumin and ferulic acid. PLLA—poly-L-lactic acid; PEG—polyethylene glycol. Included references are listed ascending in accordance to their publication date.

Curcumin		
Ref.	Type of Nerve Lesion and Animal Model	Type of Administration/Experimental Groups
Jahromi et al., 2019 [108]	Sciatic nerve injury, 10 mm gap repaired PLLA tube filled with curcumin encapsulated with chitosan nanoparticles; adult male Wistar rats	- Control without any lesion or treatment; - 8 mm nerve autograft; - 10 mm nerve gap without any following treatment; - 10 mm nerve gap repaired by PLLA conduit + fibrin gel; - 10 mm nerve gap repaired by PLLA conduit + Schwann cells; - 10 mm nerve gap repaired by PLLA conduit + nanocurcumin; - 10 mm nerve gap repaired by PLLA conduit + Schwann cells and nanocurcumin. Samples were harvested 12 weeks after surgery.
Kasmaie et al., 2019 [107]	Sciatic nerve crush injury; male Wistar rats	- Sham (control); - Curcumin during dark period (D Cur); - Vehicle (DMSO); - Curcumin during light period (L Cur); - Melatonin during light period (L Mel); - Crush injury without any treatment; - Melatonin during dark period (D Mel). Curcumin (100 mg/kg) and melatonin (10 mg/kg) were injected intraperitoneally for 4 weeks after surgery over two periods of light (9:00 a.m.) and dark (9:00 p.m.).
Caillaud et al., 2018 [106]	Sciatic nerve crush injury; male Sprague Dawley rats	- Sham group; - Saline; - Vehicle (PEG-300); - Curcumin. Curcumin was solubilized in polyethylene glycol (PEG) 300 at a concentration of 35 mg/mL. Local administration though a mini-osmotic pump. Continuous delivery of curcumin at a dose of 0.2 mg/day for 4 weeks.
Moattari et al., 2018 [104]	End-to-end repair on sciatic nerve; adult male Wistar rats	- End-to-end repair; - End-to-end repair + curcumin 100 mg/kg per day intraperitoneally administrated daily for 4 weeks after surgery; - End-to-end repair + membrane around the injured nerve; - End-to-end repair + membrane + curcumin 100 mg/kg per day, intraperitoneally administrated daily for 4 weeks after surgery. Samples were harvested 8 weeks after surgery.
Sang et al., 2018 [105]	Sciatic nerve crush injury; male Sprague Dawley rats	- Curcumin; - Curcumin + LY294002; - Curcumin + NGFshRNA; - Negative controls. Samples were harvested 4 weeks after treatment.
Zhao et al., 2017 [103]	Sciatic nerve crush injury; Male Sprague Dawley rats	- Curcumin; - PD98059 (30 mg/kg/day, i.p.) + curcumin; - IGF–1 (0.5 mg/kg/day, i.v.) + curcumin; - Negative control. Curcumin was dissolved in DMSO (20 mg/mL) at 100 mg/kg/day and intraperitoneally administrated daily for 60 days.
Ma et al., 2013 [100]	Sciatic nerve crush injury; young adult male Sprague Dawley rats	- 50 mg/kg curcumin; - 100 mg/kg curcumin; - 300 mg/kg curcumin; - 100 µg/kg mecobalamin (positive group); - Saline (vehicle group). Curcumin and mecobalamin were dissolved in saline and intraperitoneally administrated daily for 4 weeks starting immediately after nerve injury.
Mohammadi., 2013 [109]	Sciatic nerve injury, 10 mm gap repaired with a silicone tube; male Wistar rats	Silicone tube filled with 10 µL curcumin (5 mg/mL) dissolved in olive oil.
Noorafshan et al., 2011 [102]	A 30 s crush injury induced by a serrated hemostat; adult female Sprague Dawley rats	- Control animals received daily gavage of vehicle (olive oil); - Sham-operated rats (daily gavage of the vehicle); - Nerve crush, treated with the vehicle; - Nerve crush, treated with curcumin (100 mg/kg/day) dissolved in olive oil. Samples were harvested 28 days after injury.
**Ferulic acid**		
Zhu et al., 2016 [110]	Sciatic nerve crush injury; Sprague Dawley rats	- Ferulic acid 50 mg/kg, intraperitoneal injection daily for 7 days after the injury; - Saline (PBS) injection at the same time points.
Lee et al., 2013 [111]	15 mm sciatic nerve gap repaired with silicone rubber tube filled with ferulic acid; adult Sprague Dawley rats	- Saline (controls); - 5 μg/mL ferulic acid; - 25 μg/mL ferulic acid.

**Table 7:** Lignans: Table showing the experimental method (type of nerve injury and animal model), the dose, and method of administration of flaxseed oil and sesame oil.

Flaxseed Oil		
Ref.	Type of Nerve Lesion and Animal Model	Type of Administration/Experimental Groups
Danial et al., 2020 [113]	Sciatic nerve crush injury; male Sprague Dawley rats	- No injury; - Negative control: daily oral administration of distilled water (10 mL/kg body weight per day); - Experimental group: administered with flaxseed oil (1000 mg/kg body weight per day); - Positive control: administered with mecobalamin (130 µg/kg body weight per day) using esophageal feeding tube for 28-day post-operation period.
**Sesame Oil**		
Hsu et al., 2016 [114]	Sciatic nerve crush; male SPF C57BL/6 mice	- Sham group; - Negative control (injury without treatment); - 0.5 mL/kg of sesame oil for 6 days after injury; - 1 mL/kg of sesame oil for 6 days after injury; - 2 mL/kg of sesame oil for 6 days after injury. Mice were fed daily with sesame oil using an ingestion tube.

**Table 8:** Stilbenes: Table showing the experimental method (type of nerve injury and animal model), the dose, and method of administration of resveratrol. Included references are listed ascending in accordance to their publication date.

Resveratrol		
Ref.	Type of Nerve Lesion and Animal Model	Type of Administration/Experimental Groups
Zhang et al., 2020 [123]	Crush injury of sciatic nerve; Sprague Dawley rats	- Sham; - Crush injury; - Crush injury + resveratrol (100 mg/kg) intraperitoneally injected daily for 7 days after injury; - Crush injury + 3-methyladenine (autophagy inhibitor), (50 mg/kg) intraperitoneally injected daily for 7 days after injury. Nerves were harvested 14 days after crush injury.
Revin et al., 2019 [124]	Cut of sciatic nerve; adult Wistar rats	First set of experiment: - Control nerves without electrical stimulation; - Nerves stimulated with an alternating electric current of 100 imp/s for 5 min. Second set of experiment: - Sciatic nerves were injured by cutting and sutured; - Sciatic nerve injured, cut, and perfused daily in the cutting area with trans-resveratrol, 100 uL 0.1 M. Proximal and distal parts of the sciatic nerves were harvested 7 days after injury.
Ding et al., 2018 [118]	Sciatic nerve crush injury; adult male Sprague Dawley rats	- Crush-injured group; - Control vehicle 2% *v*/*v* DMSO in saline solution; - Resveratrol-treated group: 50 mg/kg intraperitoneal daily for 10 days after injury; - Resveratrol-treated group: 200 mg/kg intraperitoneal daily for 10 days after the crush injury.
Bagriyanik et al., 2014 [119]	Chronic constriction injury of sciatic nerve; male Wistar rats	- Sham group; - CCI + saline group; - CCI + resveratrol intraperitoneal injection 10 mg/kg once a day for 14 days, starting the 1st day after injury; - CCI + saline group was injected with vehicle (5% ethanol in saline).

## Data Availability

The literature was searched for using several e-sites, including PubMed, Embase, Scopus, Web of Science, and Google Scholar for work published until March 2022. Furthermore, the bibliographies of all selected articles were screened in order to find any additional relevant papers. All selected articles were peer-reviewed and were published in English. Articles that showed a relationship between peripheral nerve injury/regeneration and polyphenols were selected. Since we set the focus for this narrative review on the effect of polyphenols on peripheral nerve regeneration, only the literature that reported on the evaluation of polyphenolic compounds investigated in this specific context was included. We took all efforts for including all available and relevant articles and apologize in advance if we inadvertently missed some publications.
